# CytoJournal's move to the new platform: More on financial model to the support open-access charter in cytopathology, publication quality indicators, and other issues

**DOI:** 10.4103/1742-6413.44572

**Published:** 2008-12-16

**Authors:** Vinod B. Shidham, Martha B. Pitman, Richard M. DeMay, Barbara F. Atkinson

**Affiliations:** Medical College of Wisconsin, Milwaukee, WI, USA; 1 Harvard Medical School, Boston, MA, USA; 2 University of Chicago, Chicago, IL, USA; 3 Kansas University Med Center, Kansas City, KS, USA

As discussed during the Nov, 2008 editorial board meeting (corresponding with the 56^th^ Annual American Society of Cytopathology (ASC) Scientific Meeting at Orlando, FL, USA), CytoJournal has moved to a new platform (MedKnow Publications, http://www.medknow.com/). Currently most of the aspects of the move are complete. PDFs of all the articles published previously in CytoJournal before June 2008[1–86] will continue to be available on the new platform under the same URL of www.cytojournal.com (in the top blue bar, click on ‘Browse articles’ http://www.cytojournal.com/browse.asp) or click on ‘Search CytoJ Articles’ http://www.cytojournal.com/search.asp). They will also be available through other sites including PubMed (http://www.ncbi.nlm.nih.gov/PubMed/). All the articles published after the June, 2008 move would be available free under open access charter in HTML format. The PDFs will be available free to all Cytopathology Foundation members (http://www.cytojournal.com/CFMember.asp) and to the members of various organizations joining the ‘CytoJ OA steward’ program (http://www.cytojournal.com/OASteward.asp).

## ‘CYTOJOURNAL OPEN ACCESS STEWARD’ PROGRAM

In addition to the continued high standard of CytoJournal publication quality and the excellent online submission and online peer-review system, this platform offers many additional features. With the completion of this move to the new platform, Cytopathology Foundation Inc. (http://www.cytopathology-foundation.org/) (CF) and CytoJournal can extend numerous benefits of open-access charter in Cytopathology to encourage dissemination and sharing of scholarly Cytopathology literature. This is a significant benefit to Cytopathology as a science in particular and as information pool for evidence based information in general. As a result, the Cytopathology Foundation and CytoJournal have structured a ‘CytoJournal Open-Access Steward’ (CytoJ OA Steward) program.

*Under the* ‘CytoJ OA Steward’ *program, individual member components of any organization, society, association, department, or institution can receive FREE benefits of open access just by endorsing the open access charter of Cytopathology Foundation AT NO COST.* ‘CF open access charter’ supports free access to scholarly Cytopathology literature in CytoJournal without enforcing the *flawed practice* of *copyright transfer* on the authors. The CytoJournal authors in return agree to share the copyright under the Creative Commons Attribution License (http://creativecommons.org/licenses/by/2.0), which permits unrestricted use, distribution, and reproduction in any medium, provided the original work is properly cited.

## EDITORIAL BOARD [Figure 1]

**Figure 1:**
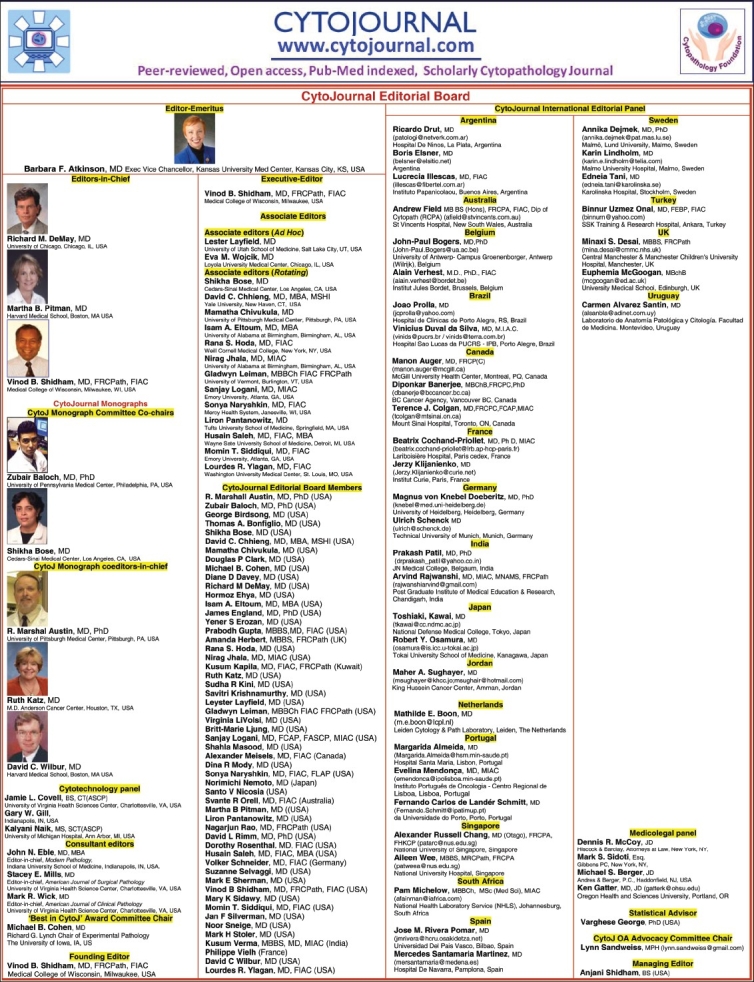
Updated CytoJournal editorial board (http://www.cytojournal.com/eb.pdf)

Because of an increase in the number of manuscript submissions, CytoJournal has updated and reorganized the editorial board, (http://www.cytojournal.com/eb.pdf) [Figure 1]. Some editorial board members who have expressed interest in contributing more time and efforts are identified as Associate Editors (AE). Designated AE of that month will complete the entire peer review cycle for all the manuscripts submitted during the corresponding month. The peer review cycle generally includes identifying qualified peer reviewers, inviting the qualified peer reviewers, and sending the manuscripts in a double blind fashion to the reviewers who have accepted the invitation. After reviewing the comments by various peer reviewers, the manuscripts would be processed further up to the stage of final evaluation by the AE. The final decision regarding acceptance or rejection would be communicated to the executive editor. Depending on the complexity of the manuscript subject matter and reviewer's criticisms, the executive editor may consult other editors in chief and the editorial board members as indicated to make the final decision of acceptance, re-revision, or rejection. After final acceptance, the manuscript is moved to the publisher for completing the publication process.

The current editorial board with designated Associate Editors and other updates [Figure 1] are posted under ‘editorial board’ http://www.cytojournal.com/eb.pdf on the CytoJournal web site.

## SELF SUSTAINING FINANCIAL MODEL FOR LONG-TERM STABILITY

### CF membership program (http://www.cytojournal.com/CFMember.asp)

Cytopathology Foundation has introduced a very economical CF membership program http://www.cytojournal.com/CFMember.asp. This program is to establish a self-sustaining financial model with the opportunity for constituent beneficiaries of the open access charter in cytopathology to contribute to its long-term success. *As most of the manuscripts would be published under the CF membership benefit, this model should nullify the perceived conflict of interest about the alternative of charging all the authors for the publication of accepted manuscripts.* Although the regular annual membership fee is nominal ($50 per year), it is free during 2008 and 2009. The other option is a *full membership* at the low cost of $1,000 for life. *Full membership* has more benefits than the regular annual membership, with many more features to be added in the future.

### *CF members* receive the following benefits:

1. Annual and full members: Free access to all PDFs in CytoJournal (otherwise $25 per download, HTML is free to all).
2. Receive FREE e-mail communications with electronic Table of Contents (e-TOC) of published CytoJournal articles at selected periodicity with links for PDF downloads.
3. Publish free in CytoJournal (if submitted manuscript is acceptable after double blind peer review). (Regular Article Publication Charge (APC) of $1500 is waived for CF members).
4. 50% discount towards most of the Cytopathology Foundation publications including CytoJournal monographs and hard copy of CytoJournal.
5. Special access to ongoing programs including standing orders (for CytoJournal Monographs and paper edition of CytoJournal) with further decrease in the prices of various publications and services.
6. Other ongoing add-ons including CME on published material, on-line tests, blogs, on-line consultation club, webinars (online seminars), and other benefits. Full members have the option to certify as CF fellowship in Cytopathology after fulfilling internationally appropriate qualifying criteria including specific academic requirements and tests.

### Information about the Article Publication Charge

For any enterprise to be self-sustaining, it is crucial to establish and execute a financially vibrant model. As mentioned at the beginning of this editorial, the new platform facilitates many of these features. One of the components of this model is contribution by the readers and authors as CF members for long-term financial stability and viability of CytoJournal. In addition to this avenue, the readers, authors, and other CytoJournal well-wishers are appealed to donate through online encrypted secure process with PayPal (click ‘*Donate*’ icon on left lower side of the CytoJournal home page www.cytojournal.com).

The authors who are not CF members or do not belong to ‘CytoJournal open-access steward’ program, still have the opportunity to get all the benefits of open access charter by paying a flat APC (Article Publication Charge) of $1,500 towards the publication expense in case the manuscript is accepted after completion of a double-blind peer-review process. *This cost is comparable to and even lower than the current standard practice in ‘open access’ model of publication by other prominent publishers* including non-profit entity such as Public Library of Science [PLoS] (http://www.plos.org/journals/pubfees.html) the *Open Access Project at Public Knowledge* and also commercial publisher such as BioMed Central (http://www.biomedcentral.com/info/authors/apcfaq#howmuch). The editorial activity of the CytoJournal will continue to be provided at no cost to all the authors for all the manuscripts- accepted or rejected.

### The APC pays for many expenses mentioned below as a brief list (but not an exclusive).

- Processing of raw manuscript for high-quality online publication in various formats.
- Barrier-free, instant, global open access to the full text of the published manuscript with unprecedented exposure to the researcher's work.
- Archival of the published articles in PubMed as soon as possible.
- Organizing inclusion in CrossRef with [DOI ^®^ http://www.doi.org/] (to facilitate electronic citation in other electronically available journals).
- Developing, updating, and maintaining software for high quality of manuscript submission system.
- Overall long-term self-sustenance of CytoJournal to support open access charter in Cytopathology.

The APC of $1,500 is significantly subsidized to $500 for the individual members of ‘CytoJ OA Steward’ (open to any department, institution, society, association, and other organizations endorsing Open Access charter of CytoJournal without any cost to the individual organization). For more details, please browse the CytoJournal website under different areas including CF membership benefits, CytoJ OA steward program benefits, etc.

## INDICATORS TO ASSES AND COMPARE THE QUALITY OF PUBLICATIONS

CytoJournal, being an online journal, has *many benefits of monitoring quality indicators in real time on its web site itself * (http://www.cytojournal.com).

### 1. Most popular articles

The ten most popular articles are displayed by clicking on ‘Most popular articles’ on left hand side.

### 2. Article access statistics

**Figure 2:**
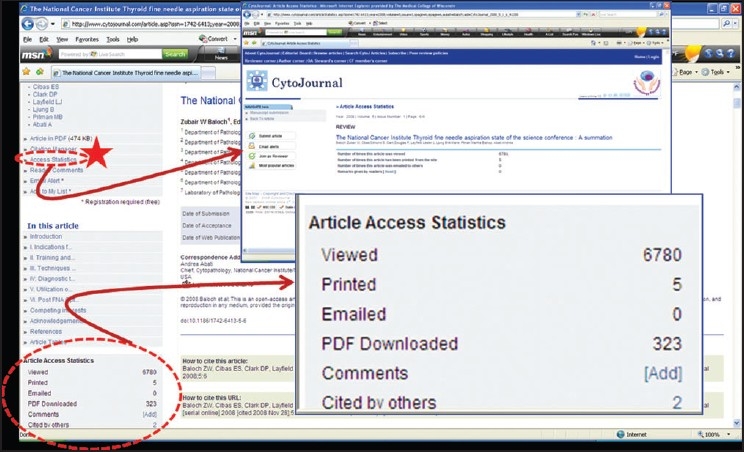
Article access statistics for individual articles in real-time

Article access statistics for individual articles is available on line in real-time while reviewing each article in HTML (left hand side) [Figure 2].

### 3. Other additional quality indicators

Other additional quality indicators, some in real-time as a FREE resource, are also available. CytoJournal falls in the range of other peer-reviewed Cytopathology scholarly journals and demonstrates indices corresponding with high quality.

Including *Impact factor* which is available through a commercial resource Thomson Reuters, http://thomsonreuters.com/about/ (previously ISI), there are many other *quality indicators* from a variety of *third party * open and FREE resources such as *Google Scholar* (http://scholar.google.co.uk/), *SCImago research group* (http://www.scimagojr.com/index.php), *Index Copernicus* (http://journals.indexcopernicus.com/masterlist.php?litera=aandstart=0andskok=30), and Harzing.com to mention a few.

### 3A. Commercially available quality indicators

*Impact factor* - Usually abbreviated as IF and provided by the *commercial resource* Thomson Reuters http://thomsonreuters.com/about/ previously called *The Institute for Scientific Information ® (ISI* ). It is one of the measures of the citations and historically had been the important indicator in the non-internet era. Although it has many limitations,[87] it is traditionally used as a tool to compare the importance of particular journal in a particular field.

IF is calculated from the data gathered over three-year period. It is calculated on the basis of the average number of citations (in year X for which IF is calculated) by all journals to those articles published in the two preceding years by journal ABC.[88]

For example, the 2008 IF of a journal ABC would be calculated as follows:

*N* = the number of citations during 2008 by various journals to all the articles published in 2006-7 by the journal ABC.

*P* = the number of "citable articles" published in 2006-7 by the journal ABC.

2008 *impact factor* for the journal ABC = *N* /*P*

(2008 IF would be available in 2009 as it could not be calculated until all of the 2008 publications had been considered.)

Although the official IF for CytoJournal will be available in near future, *the roughly calculated unofficial impact factor [based on citation data from Google scholar* http://scholar.google.co.uk/ *using the above formula to calculate IF] is comparable to other Cytopathology journals*.

### 3B. *Real-time* quality indicators available through FREE online open resources

As mentioned above, currently there are many other real-time, more flexible quality indicators allowing comparison between different journals in open system. These indicators not only provide quality evaluation of a particular journal, but also permit quantification of isolated articles and individual authors for comparative evaluation. *The, department chairs-leaders, academic institutions, readers, and authors have FREE access to monitor and quantify individual articles, journals, and authors for promotion and other academic purposes.*

**i. *h-index*:** Sometimes referred to as the *Hirsch index* or *Hirsch number*, quantifies scientific productivity and impact by the author. An author with an index of *h* has published *h* articles. Each of these *h* articles has been cited by other scholars at least *h* times (http://en.wikipedia.org/wiki/Hirsch_number#cite_note-2). This index is useful for *comparing* authors *working in the same field* as citation patterns differ widely among different fields. This index is ideal for promotion and comparison of different faculty in the same area of expertise.

**ii. *Index Copernicus*** (http://journals.indexcopernicus.com/info.php) provided through the freely available web site, which also explains how the index is generated (http://journals.indexcopernicus.com/info.php) with opportunity to compare with other journals from the data about other journals under the ‘Master List’ http://journals.indexcopernicus.com/masterlist.php?litera=aandstart=0andskok=30

**iii. *SCImago research group*** (http://www.scimagojr.com/index.php) has numerous features including SJR, H-index, and other quality indicators for comparison of various journals as a free resource.

**Figure 3:**
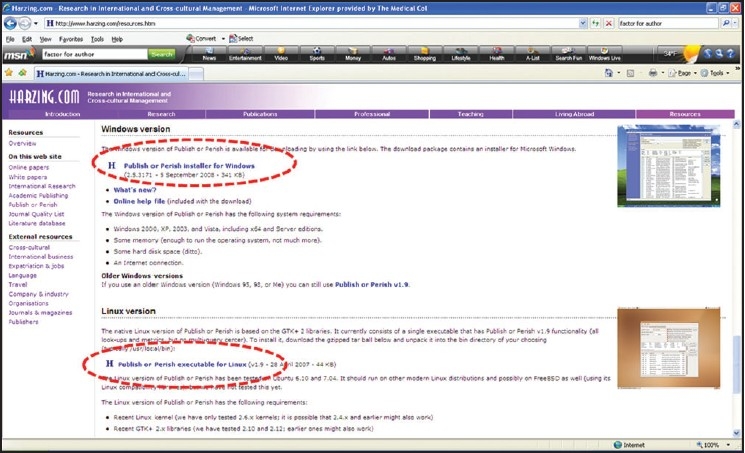
Free download of ‘Publish or perish’ software from Harzing.com http://www.harzing.com/resources.htm (Scroll to the bottom of the page)

**Figure 4:**
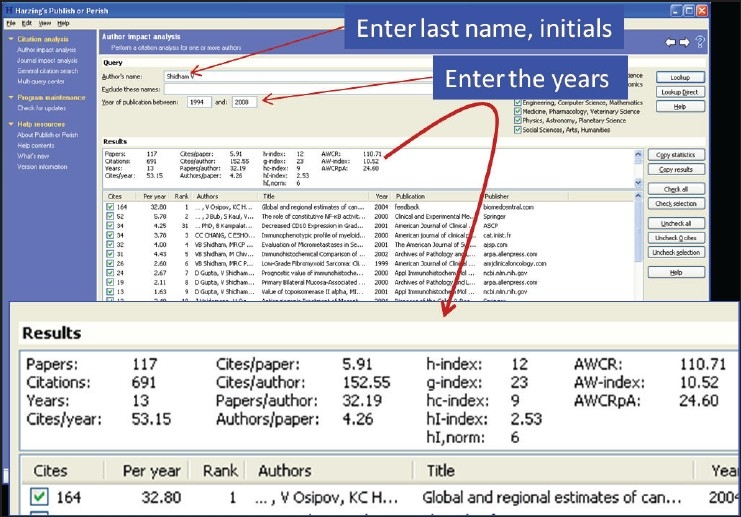
Author impact analysis performed with FREE ‘Publish or perish’ software

**Figure 5:**
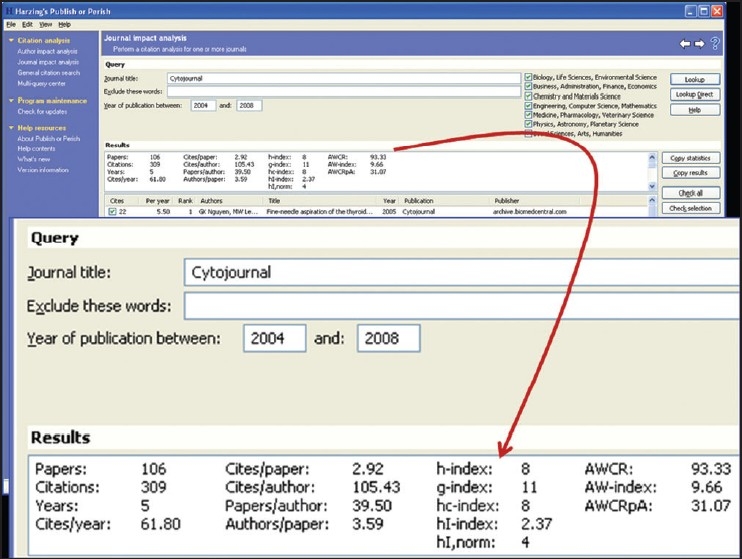
Journal impact analysis performed with FREE ‘Publish or perish’ software

**iv. *Harzing.com*** provides free software for *citation analysis* of individual authors and journals. To download this software visit http://www.harzing.com/resources.htm and click on ‘Publish or Perish installer for Windows’ (or for Linux) towards the bottom of this page by scrolling downward [Figure 3]. Citation analyses can be performed for individual authors [Figure 4] or journals [Figure 5] in 2 minutes based on online real-time *Google scholar* data http://scholar.google.co.uk/

## CYTOJOURNAL OPEN ACCESS ADVOCACY

**Figure 6:**
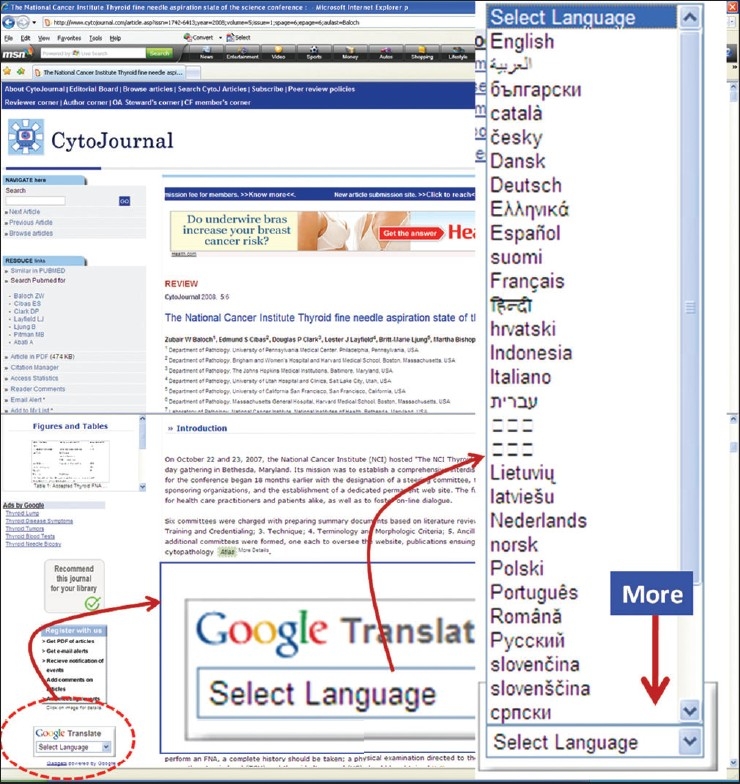
Now the articles could be translated instantly in many languages around the world. Select the language by scrolling 'Google Translate' box on lower left hand side

CytoJournal strongly recommends that all the editorial board members, reviewers, authors, readers, and other well-wishers to participate in the ‘CytoJournal Advocacy’ and ‘CytoJ open access scholar’ program (http://www.cytojournal.com/cytooascholar.asp). An open access related poster/flyer may be downloaded from http://www.cytojournal.com/CytoJ-OpenAccess.pdf and e-mailed to your colleagues. Please post/circulate those in your department, institution, societies, and conferences, in order to communicate the benefits of this open access charter of CytoJournal. The current platform in addition to the very important feature of worldwide open access benefit has *instant translation feature with translation into many languages around the world* [Figure 6].

We look forward to the continued support and active participation by all readers, authors, peer reviewers, and others considering the contents in CytoJournal as a resource of evidence based scientific literature. On behalf of CytoJournal, we thank Cytopathology Foundation Inc. (CF), its secretary/legal advisor Atty. John K. Bartosz, JD, LL.M (The Kingsbury Firm, LLC), and managing editor of CytoJournal- Anjani Shidham for their invaluable contribution of providing expertise in various activities of CytoJournal.
